# How stable is repression of disallowed genes in pancreatic islets in response to metabolic stress?

**DOI:** 10.1371/journal.pone.0181651

**Published:** 2017-08-09

**Authors:** Katleen Lemaire, Mikaela Granvik, Anica Schraenen, Lotte Goyvaerts, Leentje Van Lommel, Ana Gómez-Ruiz, Peter in ‘t Veld, Patrick Gilon, Frans Schuit

**Affiliations:** 1 Gene Expression Unit, Department of Cellular and Molecular Medicine, KU Leuven, Leuven, Belgium; 2 Pôle d’Endocrinologie, Diabète et Nutrition, Institut de Recherche Expérimentale et Clinique, Université Catholique de Louvain, Brussels, Belgium; 3 Department of Pathology, Vrije Universiteit Brussel, Brussels, Belgium; International University of Health and Welfare School of Medicine, JAPAN

## Abstract

The specific phenotype of mature differentiated beta cells not only depends on the specific presence of genes that allow beta cell function but also on the selective absence of housekeeping genes (“disallowed genes”) that would interfere with this function. Recent studies have shown that both histone modifications and DNA methylation via the de novo methyltransferase DNMT3A are involved in repression of disallowed genes in neonatal beta cells when these cells acquire their mature phenotype. It is unknown, however, if the environmental influence of advanced age, pregnancy and the metabolic stress of high fat diet or diabetes could alter the repression of disallowed genes in beta cells. In the present study, we show that islet disallowed genes—which are also deeply repressed in FACS-purified beta cells—remain deeply repressed in animals of advanced age and in pregnant females. Moreover, the stability of this repression was correlated with strong and stable histone repression marks that persisted in islets isolated from 2 year old mice and with overall high expression of *Dnmt3a* in islets. Furthermore, repression of disallowed genes was unaffected by the metabolic stress of high fat diet. However, repression of about half of the disallowed genes was weakened in 16 week-old diabetic db/db mice. In conclusion, we show that the disallowed status of islet genes is stable under physiological challenging conditions (advanced age, pregnancy, high fat diet) but partially lost in islets from diabetic animals.

## Introduction

Insulin is best known for its capacity to limit a rise in blood glucose after a meal and for the hyperglycemia in patients with diabetes when it is present in insufficient quantities. But, as patients treated with insulin know from experience, too much insulin at any given moment is an acute health hazard as it causes hypoglycemia that interferes with normal brain function. These two important aspects of insulin—its beneficial effect after a meal and its potential toxicity when present during inappropriate circumstances—require pancreatic beta cells that respect this duality [1]. Indeed, as we have proposed before [1,2] both regulatory aspects can be explained by a beta cell phenotype with two faces. The first has been thoroughly studied and depends on the presence of a large set of beta cell specific proteins. The second, however, was only recently discovered and more “hidden” as it is established by the selective absence in beta cells of a small set of housekeeping genes [3–5]. Examples of beta cell proteins that help to shape the first face are many. Some act as transcription factors that facilitate the expression of insulin mRNA [6] or as proteins that mediate the processing and folding of newly formed proinsulin [7] and the crystallization of newly formed insulin with zinc [8]. Another set of beta cell proteins of the first face ensures that beta cells, when stimulated during a meal, respond with a physiologically appropriate rate of exocytosis of insulin granules, precisely the amount needed to bring blood glucose back to normal. As has been reviewed recently [2,9], this set of proteins is responsible for the correct measurement of blood glucose and the signal transduction pathways needed for regulated exocytosis of insulin granules. Much less is known, however, about the second “hidden” face in which genes that are used for housekeeping functions in other tissues are selectively repressed in islets [2–5]. The best characterized example is repression in normal beta cells of the lactate/pyruvate transporter MCT1 which is encoded by the *Slc16a1* gene. On the one hand this absence explains why glucose metabolism in normal beta cells is so aerobic [10,11]. On the other hand, inappropriate insulin release occurs when this repression fails. Indeed, when MCT1 is present in beta cells, lactate and pyruvate can enter the beta cell during physical exercise [12], and interfere with the normal glucose sensing machinery so that insulin is secreted when blood glucose is low. Failure of repression of MCT1 was observed in a rare genetic disease called exercise-induced hyperinsulinism [13] and the same phenotype was described in transgenic mice with forced expression of *Slc16a1* in beta cells [14].

As repression of islet disallowed genes seems crucial for normal beta cell function, several studies have addressed the important question via what mechanism this type of repression is established. Although our understanding is still fragmentary, two major epigenetic mechanisms are identified: histone modifications and DNA methylation. A repressive chromatin marker -H3K27me3-, which is established during tissue maturation in the early postnatal period, was observed in a few selected disallowed genes [5] but also seen in a genome wide analysis of activation and repression of specific genes when beta cells mature [15]. In subsequent experiments RING1B, a polycomb protein, was identified as one of the molecular components that mediated these epigenetic changes [16]. More recently it was proposed that the de novo DNA methyltransferase DNMT3A plays an important role in mediating epigenetic gene repression during beta cell maturation [17]. Beta cell-specific deficiency of this enzyme causes maturation defects with both a poor glucose stimulated insulin secretion during physiological stimulation and inappropriate basal insulin secretion between meals [17].

Presently, little is known about the stability of the repressed state of disallowed genes in the adult mature beta cell population of mice that age or are fed a high fat diet, two well-known diabetes risk factors. In the current study we have addressed three new questions. First, are the gene repression data we collected thus far in isolated islets representative for beta cells? Second, what is the stability of islet-specific repression when we consider major environmental factors like advanced age, high fat diet or pregnancy? Third, what is the influence of ageing on epigenetic phenomena like histone H3 methylation and *Dnmt3a* expression?

## Material and methods

### Mice and isolation of islets, alpha cells, beta cells and other mouse tissues

For ageing, high fat diet and pregnancy studies, islets and tissues were isolated from C57Bl/6JRj mice (Janvier, Le Genest-Saint-Isle, France) at different ages as indicated in the figure legends. For the isolation of alpha and beta cells GYY and RIPYY mice (between 5 and 16 months of age) expressing EYFP under the glucagon or rat insulin promoter, respectively were used [18]. To test the influence of diabetes on the repression of disallowed islet genes, we compared mRNA signals from islets isolated from 16 week old db/db mice BKS(D)-Lepr^db^/JOrlRj strain (Janvier, Le Genest-Saint-Isle, France) and control (db/+) mice. To test the effect of pregnancy on islet disallowed genes we isolated islets from 12 week old females. The high fat diet (45% fat, 35% carbohydrate, 20% protein) (Research Diet,NJ, USA), or a regular diet (9% fat, 58% carbohydrate, 33% protein) (Ssniff, Soest, Germany) was started at age 6 weeks and maintained for 16 weeks before mRNA analysis in isolated islets. Intraperitoneal glucose tolerance test (IPGTT) and glucose stimulated insulin release (GSIS) were not performed on the same mice as animals used for mRNA expression analysis, but in a parallel study with wild type mice bred from our in house (C57Bl/6JRj background) colony. In the parallel high fat diet study, we used low fat diet (10% fat, 70% carbohydrate, 20% protein) (Research Diet NJ, USA) as a control, and mice were put on the specific diet at the age of 3 weeks for 15 (IPGTT) or 20 (GSIS) weeks. Food and drinking water were available ad libitum. Mouse islets were isolated as previously described [8], with minor changes for the db/db mice, which needed 4.15’ digestion at 37°C instead of 3.30’. Islets were immediately used for RNA isolation or chromatin immunoprecipitation. Tissues were washed with PBS, snap frozen in liquid nitrogen and stored at -80°C till use. Alpha and beta cells were FACS purified and purity assessed by fluorescence microscopy was ~99%. Cells were immediately used for RNA isolation.

### RNA isolation, cDNA synthesis, quantitative RT-PCR, chromatin immunoprecipitation and micro-array

Total RNA from mouse islets, purified alpha and beta cells was extracted using a kit (Absolutely RNA microprep, Stratagene, La Jolla, CA, US) and from the other tissues using TRIzol (Thermo Scientific, Gent, Belgium). cDNA was synthesized using RevertAid first strand cDNA synthesis kit (Life Technologies, Gent, Belgium). 100 or 200 ng islets, alpha and beta cells RNA and 1 μg tissue RNA was used for cDNA synthesis, depending on the concentration. For all quantitative RT-PCRs, 5 ng input RNA was used in a total reaction of 25 μl, using Absolute qPCR mix (Thermo Scientific, Gent, Belgium) in a RotorGene3000 apparatus. All primers/probes and efficiencies are available in S1 Table. Chromatin immunoprecipitation was performed as previously described [5] with minor changes: 400 ng DNA was used instead of 650 ng and elution was performed in 40 μl DNase-free water instead of 30 μl. Primer/probe details are available in S2 Table. Micro-arrays (430 2.0 arrays) were performed as described before [5]. The data files are accessible at NCBI Gene Expression Omnibus with accession number GSE24207.

### Glucose tolerance test and glucose stimulated insulin release

For a glucose tolerance test, mice were fasted for 16 hours. A glucose bolus (2.5 mg/g BW) was administered intraperitoneally for the high fat diet study, or oral for the pregnancy study and blood glucose levels were followed during 2 hours using AccuCheck glucostrips (Roche, Vilvoorde, Belgium).

For glucose stimulated insulin release, triplicates of 5 size matched, hand-picked islets (1 big, 2 medium, 2 small) were incubated for 1 hour at 37°C in HEPES Krebs buffer containing 0.5% BSA and 5 mM (G5) or 20 mM (G20) glucose. N is the amount of independent mice analyzed (one independent sample = average of triplicates of 5 size matched islets per condition). Following incubation, insulin was quantified using ELISA (Crystal Chem Inc, Illinois,USA).

### Genomic DNA isolation and dot blot analysis

Genomic DNA from mouse tissues was isolated using a kit (GenElute Mammalian Genomic DNA Miniprep Kit,Sigma,) and was denaturated for 5’ at 96°C and spotted in a 2-fold serial dilution (100–50 ng) on a Hybound nylon positively charged membrane (Genescreen Plus, Perkin Elmer, Zaventem, Belgium) using a dot-blot apparatus. The blotted membrane was rinsed with 2x SSC, air dried and UV-cross linked at 120000 μJ/cm^2^ (UV 1000 CrossLinker). The membrane was blocked in TBS-Tween (0.5%), and total DNA methylation was detected using a 5-mC antibody (BI-MECY-0050, 1/1000; Eurogentec, Liège, Belgium). Signals were detected using ImageQuant LAS4000 (GE Healthcare, Diegem, Belgium)

### Data analysis and statistics

Quantitative RT-PCR was normalized for *ActB* expression, which is stable under the used conditions (S1, S2, S3 and S4 Figs). *Student t-*test with a multiple comparison correction (Bonferroni) was used for statistical analysis: * p<0.05; ** p<0.01; *** p<0.001. Expression patterns during ageing were analyzed using quantitative RT-PCR data with Multiexperiment Viewer (MeV) [19].

### Study approval

All experiments with laboratory animals were approved by the committee for animal welfare at the KU Leuven (P124/2012) and at the UC Louvain (2014/UCL/MD/016).

## Results

### Islet disallowed genes are deeply repressed in FACS purified beta cells

**Fig 1:**
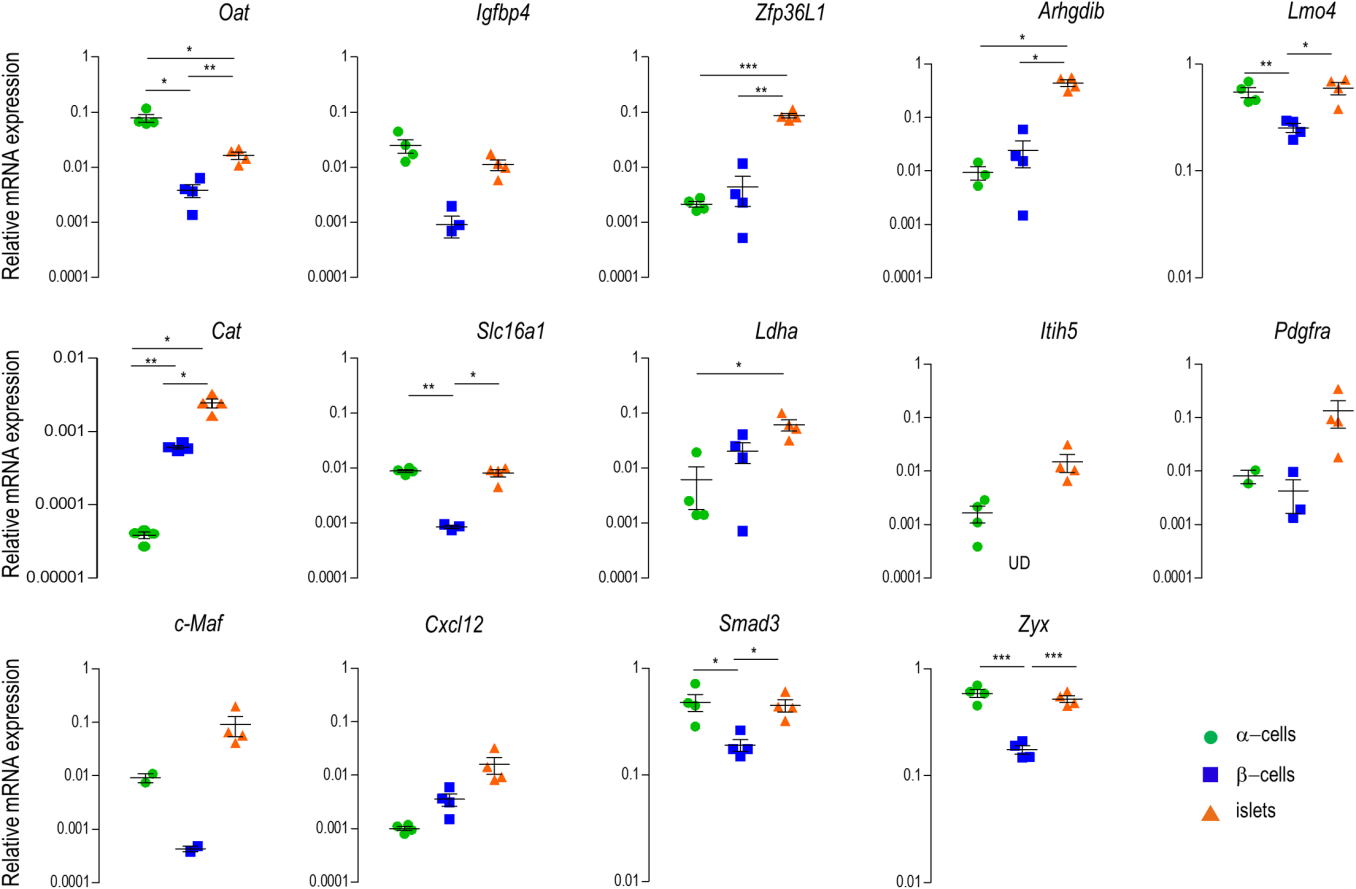
Analysis of mRNA expression of islet disallowed genes in freshly collagenase-isolated islets, compared with FACS purified alpha and beta cells. Data are normalized for *ActB* and expression in liver (from 12 weeks old mice) is set as 1, since liver has a high and robust expression of the islet disallowed genes, making it immediately clear that these genes are very low expressed in islets. Data represent mean±SEM, N≥3, Statistical analysis: *student t*-test with multiple comparison correction (Bonferroni). UD, under detection limit.

On the basis of genome wide mRNA expression studies using two types of microarray platforms we identified a signature of 14 genes that are significantly repressed in islets compared to all other tested tissues [1,5]. Since pancreatic islets are composed of several cell types, which are likely to have different expression patterns, we next compared mRNA signals of each of the 14 signature genes in whole islets with FACS purified alpha and beta cells. The contamination of beta cells in the alpha cell preparations and vice versa was in the order of magnitude of 1 percent as estimated from the measurement of pancreatic islet hormones (S1A Fig). For all the analyzed islet disallowed genes—*Arhgdib*, *Cat*, *Cxcl12*, *Igfbp4*, *Itih5*, *Ldha*, *Lmo4*, *Maf*, *Oat*, *Pdgfra*, *Slc16a1*, *Smad3*, *Zfp36l1*, *Zyx*—the observed expression in FACS purified beta cells was found to be equally or even more repressed compared to the expression in whole islets (Fig 1). Also for alpha cells, most genes of the panel had an expression profile which is similar as that in islets or beta cells, however, for two genes (*Oat* and *Cat*), mRNA expression in alpha cells was both significantly higher (*Oat*) or lower (*Cat*) with expression in beta cells and whole islets (Fig 1). Together our data indicate that mRNA identification of islet disallowed genes is confirmed (even at a deeper level of repression) in beta cells for each of the 14 genes of the signature. For the next studies we therefore used whole islets to analyze the effect of environmental factors such as advanced age, high fat diet and pregnancy on the repression level of these disallowed genes in isolated islets.

### Robust repression of islet disallowed genes during ageing

**Fig 2:**
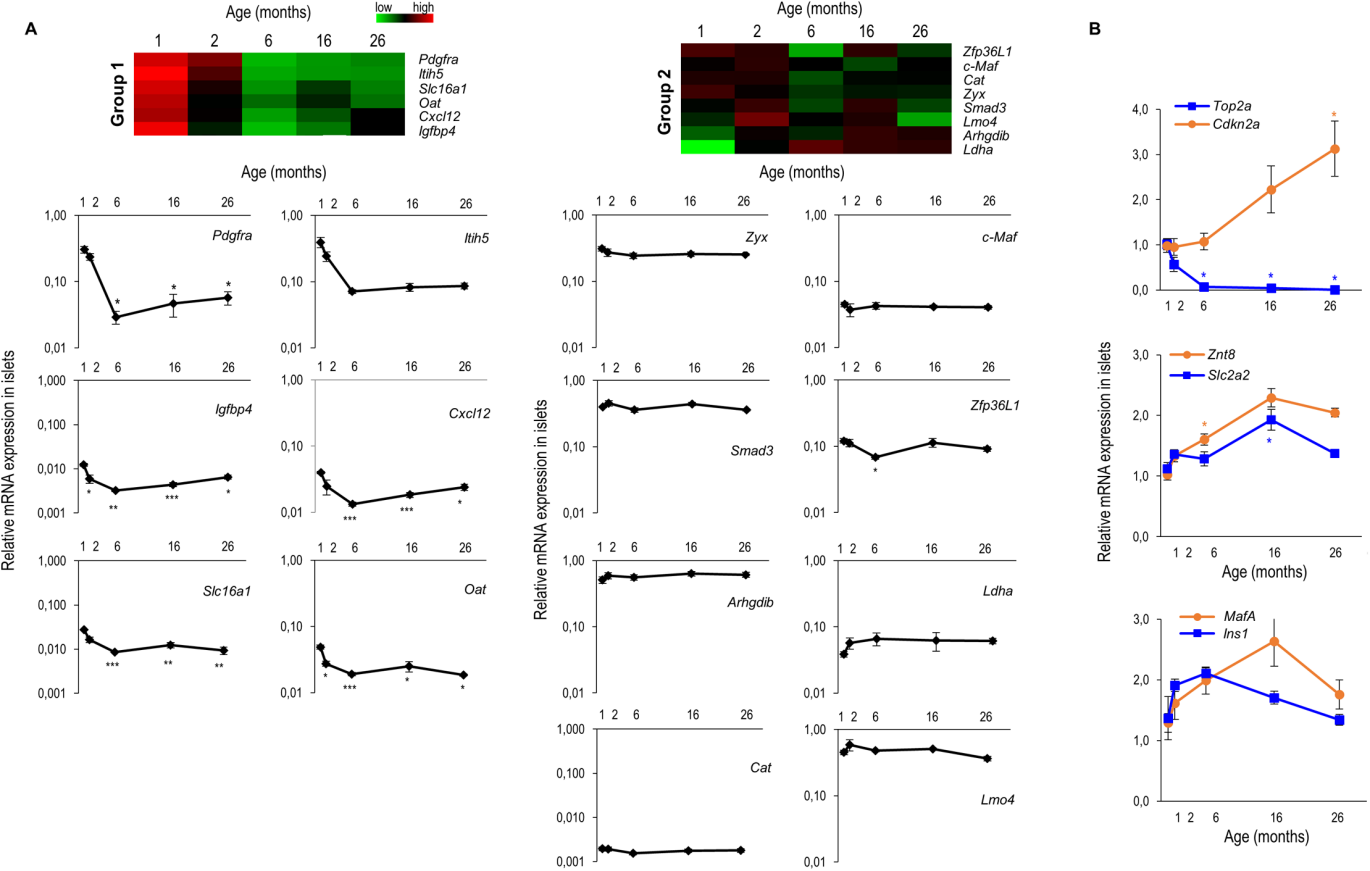
Effect of age on the expression of disallowed islet genes. **(A)** Upper panel heat map of the expression signals of the signature of 14 islet specifically repressed genes in islets isolated from mice of different ages (1, 2, 6, 16 and 26 months) using Multiexperiment Viewer [19]. Group 1 has six genes in which repression matures between months 1 and 6 of life and is then maintained; group 2 has eight genes which repression is independent of age. Lower panel, mRNA expression levels of the different disallowed genes of group1 and group2. Quantitative RT-PCR data are normalized for beta actin, and expression level in liver (from 12 weeks old mice) is set as 1; **(B)** mRNA expression of key genes involved in proliferation/maturation and beta cell function. Quantitative RT-PCR, normalized for beta actin and expression at 1 month is set as 1. Data represent mean±SEM, N = 4. Statistical analysis: *student t*-test with multiple comparison correction (Bonferroni), compared to expression at 1 month of age.

Ageing is an important factor in the development of type 2 diabetes. To analyze if weakening of repression of the islet specific repressed genes is a factor involved in the increased incidence of type 2 diabetes development with ageing, we analyzed the effect of ageing on the repression of the 14 disallowed genes. As can be seen in Fig 2, we compared mRNA signals from disallowed genes in islets aged 1 month, 2 months, 6 months, 1 year (16 months) and 2 years (26 months). Body weight and blood glucose levels of these mice can be found in S2A Fig. The age-dependent signals of the 14 genes signature can be divided in 2 expression groups. Group 1 of the signature contains six genes whose expression is altered with age (Fig 2A), typically starting with incomplete repression at 1 month and a maturation of repression between 1 and 6 months, after which repression is maintained until advanced age (Fig 2A). Group 2 contains the remaining 8 genes in which repression appears largely unaffected by age in the window we investigated (Fig 2A). Next, we also analyzed the expression of the genes of group 1 in liver and diaphragm of mice of the same age, to exclude that the observed expression patterns in islets are a general behavior in all tissues (S2C and S2D Fig). As expected from the literature [20] expression of *Top2a* (a cell cycle protein) and *Cdkn2a* (a cell cycle inhibitor) clearly reflected the decrease of proliferating beta cells with advancing age (Fig 2B). Finally we measured four genes that are representative for the function of mature beta-cells and observe no weakening of the islet function (Fig 2B). Together, our data show that repression of all 14 genes of the disallowed gene signature is stable even at the advanced age of 2 years and for 6 genes of the signature maturation of repression can be seen during the first months of life.

### Epigenetic regulation of the repression of disallowed islet genes

**Fig 3:**
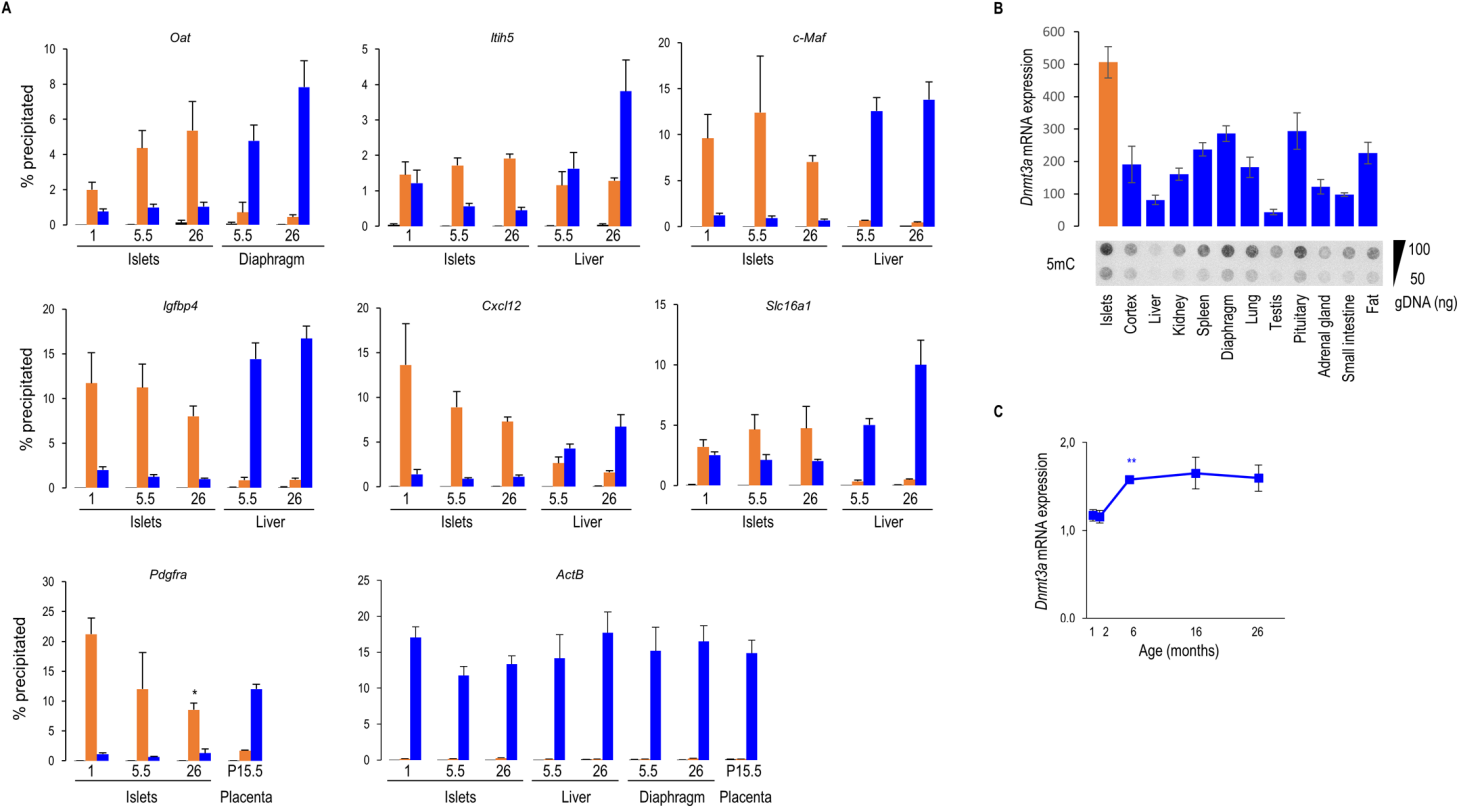
Epigenetic modifications in islets from ageing mice. **(A)** Histone H3 modifications in promoter region of islet specific disallowed genes. Data sets include IgG immunoprecipitation controls (black bars); the epigenetic activation mark, histone H3 lysine 9 acetylation (H3K9ac) (blue bars) and the repression mark, histone H3 lysine 27 trimethylation (H3K27me3) (orange bars). Pancreatic islet chromatin was isolated from mice aged 1, 5.5 and 26 months; chromatin from liver and diaphragm of mice aged 5.5 and 26 months or from placenta of 15.5 days pregnant female mice age 3 months was taken as reference tissue. For all genes, high H3K27me3 islet signals and low H3K9ac signals were observed at all ages, while in the reference tissues the opposite is seen. For all of the tested genes except *Pdgfra* the H3K27me3 signal in islets is not significantly influenced by age. As a control housekeeping gene we investigated the beta actin promoter, which is active in all tissues: high H3K9ac signals and low H3K27m3 signals were measured in all tested conditions (bottom right panel). Data represent mean±SEM, N = 3. Statistical analysis: *student t*-test with multiple comparison correction (Bonferroni). **(B)** Upper panel, mRNA expression of the de novo methyltransferase *Dnmt3a* in different mouse tissues (12 week old mice) measured via Affymetrix, 430 2.0 arrays. Lower panel, DNA methylation measured via total 5-methylcytosine (5mC) levels in different male mouse tissues (12 week old mice); **(C)** mRNA expression of *Dnmt3a* during ageing, measured via quantitative RT-PCR. Data represent mean±SEM, N = 4. Statistical analysis: *student t*-test with multiple comparison correction (Bonferroni), compared to expression at 1 month of age. Data are normalized for *ActB* expression.

In previous research we proposed that a repressive histone H3 epigenetic mark mediates the islet-specific repression of two disallowed genes *Slc16a1 (Mct1)* and *Ldha* [5]. In the previous paragraph we observed that the repression of some of the disallowed genes increased with age (group 1) and we wondered if this deeper repression correlated with the repressive histone H3K27me3 mark. Therefore, we tested the age-dependency of the well-established epigenetic marks—H3K9ac (activation) and H3K27me3 (repression)–in all genes of group 1 and c-Maf (group 2) at three different ages: 1, 5.5 and 26 months. As can be seen in Fig 3 even at the advanced age of 2 years, the islet chromatin shows a robust H3K27me3 repression mark, while the H3K9ac activation marks are very low as compared to the control tissues. Although some fluctuations in function of age were noticed, no correlation could be detected between H3K27me3 signals at 1 and 5.5 months of age and the deeper repression of group 1 genes in islets of 6 months old mice (Fig 2A). Together, these data show that the epigenetic histone repression mark on disallowed genes is very stable with ageing, but cannot explain the fine-tuning of the repression. Besides histone modifications, also DNA methylation via DNMT3A, a de novo DNA methyltransferase, has been suggested to play a role in the repression of some of the disallowed genes in islets of neonatal mice [17], but it is unknown at this moment whether DNMT3A in islets is also present during adult life. To examine this question, we quantified *Dnmt3a* expression signals in mouse pancreatic islets and compared them with expression signals in a panel of other mouse tissues (Fig 3B, upper panel). Expression of *Dnmt3a* in islets was clearly higher than in the other analyzed tissues and this correlated well with high total DNA methylation in islets, as measured via total 5-methylcytosine signal (Fig 3B, lower panel). The expression of *Dnmt3a* increased with age, in an opposite pattern as the repression of disallowed genes from group 1 (Fig 3C). With the current experimental approach correlations can be made between high Dnmt3a mRNA expression in islets and stable epigenetic chromatin marks; nevertheless our data support the emerging idea [1,5,17] that histone and DNA methylation events play an important role during pancreatic beta cell maturation when islet disallowed genes are repressed.

### A high fat diet feeding has no effect on the repression of islet disallowed genes

**Fig 4:**
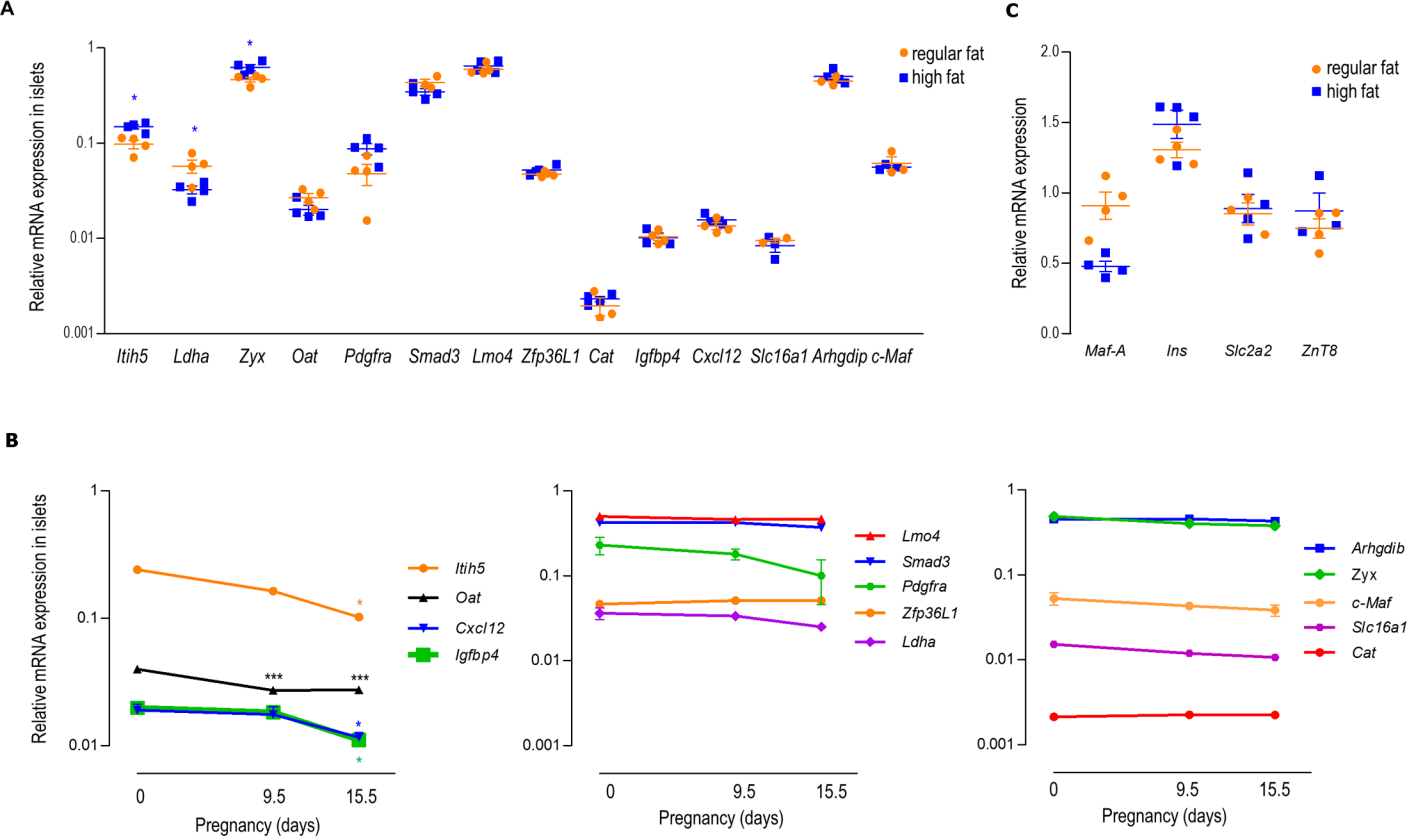
Effect of high fat diet feeding and pregnancy on the expression of disallowed islet genes. **(A)** mRNA expression level of the disallowed genes in islets of male mice (22 weeks old mice) fed a high fat diet for 16 weeks or **(B)** of female pregnant mice (pregnancy day 0–9.5–15.5) (12 weeks of age) measured via quantitative RT-PCR. Data are normalized for beta actin, and expression level in liver (from 12 weeks old mice) is set as 1; **(C)** mRNA expression of key genes involved in beta cell function, measured via quantitative RT-PCR and normalized for beta actin. Data represent mean±SEM, N = 4. Statistical analysis: *student t*-test with multiple comparison correction (Bonferroni).

Besides advanced age, a high fat diet is also considered as a risk factor for type 2 diabetes [21]. Therefore, we fed male mice for 16 weeks a high fat (45% of calories) diet, starting at 6 weeks of age, while the control mice received a regular fat (9% of calories) diet. Mice that were 16 weeks on a high fat diet gained more weight compared to their littermates on regular chow and also their blood glucose levels were increased (S3A Fig). This resulted in a decrease of ~50% of *MafA* expression in islets of mice fed a high fat diet, while expression of other genes involved in beta cell function was unaltered (Fig 4B). Under these conditions, mice became glucose intolerant (S3C Fig). Islets from mice fed a high fat diet released more insulin per islet, both under basal (5 mM) and stimulated (20 mM) glucose concentrations (S3C Fig). Since insulin content per islet was also increased in mice fed a high fat diet, insulin release as percent of insulin content was not affected by the diet (S3C Fig). Next, the expression of the 14 islet specific disallowed genes was analyzed in islets of these mice (Fig 4A). Similar as during ageing, the expression of most of the disallowed genes stayed stably repressed after a high fat diet feeding (Fig 4A). Only for two, *Itih5* and *Zyx*, of the 14 genes, the repression was slightly decreased after a high fat diet feeding. However, the expression of these two genes stayed below the expression in other tissues (in liver: *Itih5* 6x and *Zyx* 1.4x higher; in diaphragm: *Itih5* 12x and *Zyx* 4x higher, compared to islets). The expression of one gene, *Ldha*, was further decreased (Fig 4A). These data show that also after a high fat diet feeding, the repression of the islet specifically disallowed genes is very stable.

### Repression of islet disallowed genes is sustained during proliferation of islets in pregnant mice

With the advance in islet transcriptome analysis, recent interest in phenotypic changes in beta cells from pregnant mice has grown [22,23]. During the second week of pregnancy, a wave of proliferating islet cells is observed [24], resulting in an increased beta cell mass. Preceding these changes, a sharp peak of cell cycle gene expression is measured at pregnancy day 9.5 (P9.5) [25]. Therefore, we measured the expression signal of disallowed genes at two time points during pregnancy, P9.5 and P15.5. Under these conditions, body weight was increased as expected and blood glucose levels were unchanged (S4A Fig). In contrast to a high fat diet feeding, pregnancy did not result in a clear glucose intolerance (S4B Fig), while glucose stimulated insulin release per islet was significantly increased at P15.5 and also insulin content per islet was increased (S4C Fig). For the 14 disallowed genes, the repression was not decreased during pregnancy (Fig 4B). On the contrary, the repression of four out of 14 signature genes (*Itih5*, *Oat*, *Cxcl12* and *Igfbp4*) significantly increased at one or two time points of pregnancy (Fig 4C). Together, these data show that repression is well preserved during proliferation in pregnancy.

### Partial loss of repression of islet disallowed genes in db/db mice

**Fig 5:**
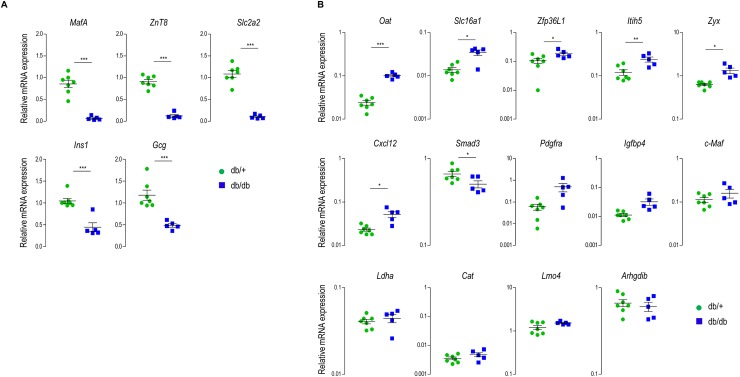
Expression of the islet disallowed genes in the diabetic db/db mouse model. **(A)** mRNA expression of key genes required for beta cell function and expression of the hormones insulin and glucagon. Quantitative RT-PCR, normalized for beta actin and expression in the db/+ mouse is set as 1. **(B)** mRNA expression level of the 14 disallowed genes in islets of male diabetic db/db mice and control db/+ mice. Quantitative RT-PCR data are normalized for beta actin, and expression level in liver (from 12 weeks old mice) is set as 1; Data represent mean±SEM, N = 7–8. Statistical analysis: *student t*-test.

In various different physiological challenging circumstances (age, diet, pregnancy) a robust repression of disallowed islet genes was measured. But would the disallowed gene signature also be stable after chronic hyperglycemia? To investigate this question, we used 16 week old db/db mice which are clearly hyperglycemic, both when measured at random (S5A Fig), or during an intraperitoneal glucose tolerance test (S5B Fig). As was earlier reported [26] the chronic hyperglycemia in this model results in changes in islet gene expression. Indeed, we observed a strong decrease in both insulin and glucagon mRNA signals (Fig 5A). Moreover, when compared to the control db/+ mice we measured in islets of db/db mice a significant loss of transcripts that are enriched in normal beta cells (*MafA*, *ZnT8*, *Slc2a2*). But what happens to the other phenotypic face of the beta cell phenotype? Fig 5B summarizes the result of mRNA signals of the 14 disallowed genes in this model. Significant loss of repression was observed in islets of db/db mice when compared to the control db/+ mice for six of these genes (*Itih5*, *Oat*, *Zfp36L1*, *Slc16a1*, *Zyx* and *Cxcl12*); and for two other disallowed genes (*Pdgfra* and *Igfbp4*) a similar, but not significant, trend was observed (Fig 5B). In contrast, the repression of one disallowed gene (*Smad3*) is stronger in db/db mice than in db/+ mice and the expression of the five other disallowed genes (*c-Maf*, *Cat*, *Ldha*, *Arhgdib* and *Lmo4*) was unchanged (Fig 5B). Together our data show that islets isolated from 16 week old db/db mice have lost phenotypic characteristics, not only for islet specific mRNA transcripts that are normally enriched, but also gained expression of a series of disallowed genes.

## Discussion

Since the introduction of the collagenase islets isolation technique [27], pancreatic islets have become a standard source of insulin producing beta cells which indeed are the most important cellular constituent of this tissue [28]. However, when a set of genes is repressed in beta cells but present in other islet cell types, the confounding expression signals from other cell types present in isolated islets may distort the interpretation events that take place in beta cells. This idea was illustrated by variable signals of low-Km hexokinase expression originating from contaminating acinar cells rather than beta cells [4,29]. Therefore, the cellular basis of tissue specific gene repression in pancreatic islets [3,5] deserves deeper analysis at the level of purified islet cell types. In the present study we show that all fourteen genes of the disallowed gene signature we have investigated were equally or deeper repressed in beta cells than in islets indicating that by using whole islets we underestimated the degree of repression in beta cells. Interestingly, expression for most of the disallowed genes of the investigated signature is low in alpha cells as well. One exception is ornithine aminotransferase (*Oat*) which is expressed in alpha cells like in most tissues of the reference panel, while much deeper repressed in beta cells. The mitochondrial enzyme OAT catalyzes the interconversion between ornithine and gamma glutamyl-semialdehyde, using glutamate and alpha-ketoglutarate as co-metabolites for the amino-group transfer reaction [30]. Currently there is no information available why this reaction could be inappropriate for beta cells, but perhaps it should be avoided for a tight control of polyamine biosynthesis [31]. A second difference between alpha and beta cell is catalase which is even more repressed in alpha than in beta cells. Low islet catalase expression was linked before to the vulnerability of beta cells to oxidative stress [32], but we have little understanding why this gene is so deeply repressed in alpha cells. The presence of genes with an expression in alpha and beta cells that is a magnitude lower than the expression in islets can be explained by contamination of the islet preparation by acinar cells, where the expression of the gene of interest is probably much higher compared to the beta cells. Together, we encountered no cases in the gene signature in which repression in whole islets is not supported by repression at the level of beta cells, so that our next question was the screen for changes in repression when islets were followed in function of age, diet and pregnancy.

Several environmental and metabolic factors can stress the functional beta cell and alter its phenotype. A pioneering study explored the effect of chronic hyperglycemia in vivo after partial pancreatectomy with the result that the expression of one disallowed gene, *Ldha* was upregulated [33]. We therefore wondered whether changes of the beta cell environment such as advanced age, high fat diet and pregnancy but also chronic hyperglycemia in the db/db mouse model could alter the expression of the whole disallowed gene signature. We previously showed that tissue disallowance is required and established in the postnatal period of islet maturation to achieve its phenotypic identity [5], but how this disallowance is behaving during ageing is not yet known. Therefore, we followed islet specific gene repression in islets of mice for 2 years. During ageing, beta cell proliferation is well controlled to maintain a correct functional beta cell mass [20,34,35]. We observed at the age of 6 months a clear decrease of *Top2a* expression and an increase of *Cdkn2a* –a cell cycle inhibitor- expression (Fig 2B), suggesting a decrease in beta cell proliferation. In contrast, no major changes on the expression of genes involved in beta cell function was seen (Fig 2B), suggesting no intrinsic decline in beta cell function with age. Our observations agree with recent studies in which age-dependent increase of *Cdkn2a* expression correlated with enhanced insulin secretion [36–38]. Comparable to the proliferation pattern, an age-dependent decline in expression is seen for a set of islet disallowed genes. Genes belonging to group 1 reached their deepest repression at the age of 6 months (Fig 2A), similar to the time point where the expression of *Cdkn2a* increases (Fig 2B). To know if repression of disallowed genes is linked with the degree of beta cell proliferation, further investigation is required, but our observation of islet gene repression during pregnancy, also suggest in some cases an increase in repression when proliferation is increased (Fig 4C). Although a high fat diet normally results in an increased beta cell mass [39], we did not observe an increased repression of the disallowed genes after our high fat diet treatment. However, we cannot exclude that under the present conditions, beta cell mass did not increase. In general, we observed no weakening of islet gene disallowance during ageing, suggesting that this repression is very stable. The observed stable epigenetic characteristics of high signals of histone H3K27 trimethylation and low signals of H3K9 acetylation during ageing confirm the idea of a stable repressive regulation of disallowed genes for the entire adult life span of beta cells. Furthermore, we show high and stable *Dnmt3a* mRNA levels in pancreatic islets, as opposed to most other tissues, even at advanced age. This high *Dnmt3a* mRNA levels are translated to high DNA methylation levels in islets as compared to other tissues. This observation extends those of a recent study in which DNMT3A was found responsible for the repression of lactate dehydrogenase (LDHA) and low-Km hexokinase (HK1) during the first weeks after birth, when most beta cells mature [17]. This investigation underlined the role of DNMT3A as a de novo methyltransferase during the period that the phenotype of adult beta cells is formed. Our analysis focused on mRNA expression signals following the well accepted working hypothesis that epigenetic regulation plays an important role for the mRNA copy number per cell. This approach does not exclude post transcriptional regulatory events which may further fine-tune the degree of repression of disallowed genes [3,5]. The functional beta cell mass, which is determining glucose homeostasis, is chronically influenced by obesity and pregnancy [40,41]. Feeding mice a high fat diet is a risk factor in the development of type 2 diabetes, which is a result of a reduction in beta cell mass [42] and of beta cell dedifferentiation [43]. Recently, Nishimura showed that MAFA is critical for maintenance of the mature differentiated beta cell phenotype [44] and islets of *MafA* KO mice showed a reduction in the expression of genes critical for beta cell function, but not of genes required for glucose stimulated insulin release. Moreover, also the expression of *Slc16a1*, one of the disallowed genes, was increased in this mouse model. Although in our study, feeding mice a high fat diet for 16 weeks–which resulted in glucose intolerance and enhanced basal and glucose stimulated insulin release–resulted in a decrease of *MafA* expression of about 50% (S4B Fig), it did not result in a decrease in gene expression of genes involved in beta cell function nor in expression of the disallowed gene signature. In contrast, in our 16 week old db/db mice, which is a model for type 2 diabetes, we do observe a severe depletion of mRNA’s that are normally enriched in beta cells (*MafA*, *ZnT8*, *Glut2*), as previously published [45–47], together with a loss of expression of islet hormone genes (Fig 5A). Interestingly, besides repression of these islet specific genes, expression of half of the disallowed gene signature is increased in islets of these db/db mice. Possibly, this beta cell dedifferentiation may contribute to the metabolic decompensation in diabetes [43]. The insulin secretory granule zinc transporter ZnT8 is essential for the formation of insulin-zinc crystals [8]; a dramatic loss of *ZnT8* mRNA therefore explains the translucent appearance of islets of db/db mice in brightfield illumination, and very poor staining with dithizone (results not shown). The loss of *Glut2* and *MafA* in islets of db/db mice can be reversed when hyperglycemia is corrected [45,47], so it would be interesting to investigate if the derepression of the disallowed genes can also be reversed. Besides obesity, also pregnancy leads to a higher functional beta cell mass [40]. Cell cycle gene expression peaks at pregnancy day 9.5 [25], while beta cell proliferation is highest at pregnancy day 15.5 [22]. We observed that four of the fourteen disallowed genes were more repressed during pregnancy, with the highest repression at pregnancy day 15.5. It is known that during pregnancy, the islet mass increases, and this is mainly because of an increase in beta cells, indicating that the beta/alpha cell ratio increases during pregnancy [48]. Since not all disallowed genes are equally repressed in alpha and beta cells, an increase in beta/alpha cell ratio, rather than a deeper repression per cell, could be the reason for the increased repression of some of the disallowed genes during pregnancy (Fig 4C), in particular for *Oat*, which is more repressed in beta cells than in alpha cells. But also intercellular differences between different beta cells can influence the obtained results. Indeed, beta cells were previously found to be heterogeneous at the level of glucose-induced insulin release and insulin biosynthesis [49,50]; more recent work indicates that a beta cell population is in fact a mixture of fully differentiated and partially dedifferentiated cells [43].

The stable repression of the signature of disallowed genes we report here in wild type mice of old age, or taking high fat diet or during pregnancy contrast with the perturbations induced by deletion of key transcription factors such as the winged-helix transcription factor Rfx6 which is essential for islet cell development [51]. Indeed, beta cell specific deletion of *Rfx6* in 10-week old mice resulted in impaired glucose homeostasis because of disturbed insulin secretion and expression of several of the disallowed islet genes was upregulated [52]. Also the transcription factor MafA is critical for maintenance of the mature beta cell phenotype in mice [53] and, as mentioned above, deletion of *MafA* resulted in reduced beta to alpha cell ratio and upregulation of disallowed islet genes leading to dedifferentiation of beta cells, which is implicated in diabetes [43,44].

In conclusion, our data show that environmental factors such as advanced age, high fat diet and pregnancy, which were studied in non-diabetic mice, do not impair the epigenetic basis (both DNA methylation and histone modification) that represses disallowed genes in beta cells. Under physiological conditions this repression is stable in function of age, diet and pregnancy. In an experimental model of diabetes, however, the disallowed gene signature is partially lost. Future studies should assess the pathophysiological significance of this loss.

## Supporting information

S1 Table Primers and probes used for quantitative RT-PCR. (PDF)

S2 Table Primers and probes used for chromatin immunoprecipitation. (PDF)

S1 Fig Analysis of the different islet hormones in the FACS purified alpha and beta cells. mRNA expression of insulin, glucagon, pancreatic polypeptide (PPY) and somatostatin in alpha-, beta- cells, compared with whole islets, measured via quantitative RT-PCR. The contamination of beta cells in the alpha cell preparations and vice versa was in the order of magnitude of 1 percent as estimated from the measurement of pancreatic islet hormones. Contamination with delta-cells (somatostatin) is in the same range. PPY expression in the beta-cell preparation is also in the order of magnitude of 1 percent. In contrast, the expression of PPY in the alpha-cells is as high as in islets, a phenomenon that has been noticed before (Gilon P., unpublished micro array data; [54,55]) and most probably is not a result of contamination with PP-cells. **(B)** Ct values of beta actin, used for normalization of the QPCR data.
(TIF)

S2 Fig Effect of ageing on the expression of disallowed islet genes in liver (C) and diaphragm (D). **(A)** Body weight and blood glucose levels of the used mice at different ages; **(B)** Ct values of beta actin, used for normalization of the quantitative RT-PCR data; **(C, D)** mRNA expression levels of the 14 islet specifically repressed genes in liver and diaphragm of mice at 5 different ages (1–2–6–16–26 months), measured via quantitative RT-PCR. Data are normalized for beta actin and expression at 1 month is set as 1. Data represent mean±SEM, N = 4.
(TIF)

S3 Fig Characterization of mice fed a high fat diet. **(A)** Body weight and blood glucose levels of the analyzed mice after 16 weeks of a high fat diet; **(B)** intraperitoneal glucose tolerance test after 15 weeks on the specific diet, N = 8, 18 weeks old mice); **(C)** Glucose stimulated insulin release after 20 weeks on the specific diet (23 weeks old mice): release per islet (left panel), insulin content (middle panel) and release per content (right panel), G5 = 5 mM, G20 = 20 mM, N = 7; **(D)** Ct values of *ActB*, used as reference gene. N = 4. Data represent mean±SEM. Statistical analysis: *student t*-test with multiple comparison correction (Bonferroni).
(TIF)

S4 Fig Characterization of pregnant mice. **(A)** Body weight and blood glucose levels during pregnancy (pregnancy day 0–9.5–15.5), **(B)** oral glucose tolerance test, N = 4, **(C)** Glucose stimulated insulin release: release per islet (left panel), insulin content (middle panel) and release per content (right panel), G5 = 5 mM glucose, G20 = 20 mM glucose, N≥5, **(D)** Ct values of *ActB*, used as reference gene. N = 4. Mice 12 weeks of age. Data represent mean±SEM. Statistical analysis: *student t*-test with multiple comparison correction (Bonferroni).
(TIF)

S5 Fig Characterization of db/db mice. **(A)** Body weight and blood glucose levels of 16 weeks old db/db and control db/+ mice, N = 8. **(B)** Intraperitoneal glucose tolerance test at the age of 15 weeks, N = 8. **(C)** Ct values of *ActB*, used as reference gene. N = 7–8. Data represent mean±SEM. Statistical analysis: *student t*-test.
(TIF)
