# Supplementary material for: How stable is repression of disallowed genes in pancreatic islets in response to metabolic stress?
Source: PLoS One. 2017 Aug 9;12(8):e0181651. doi: 10.1371/journal.pone.0181651 (PMC5549890; doi:10.1371/journal.pone.0181651)
Supplement: S1 Table — (PDF) [file pone.0181651.s001.pdf]

Gene	Forward primer	Conc. (nM)	Reverse primer	Conc. (nM)	Probe(6-FAM)-(TAMRA)	Conc. (nM)	Eff.
actb	AGCCATGTACGTAGCCATCCA	900	TCTCCGGAGTCCATCACAATG	900	TGTCCCTGTATGCCTCTGGTCGTAC	50	0.98
Arhgdib	TACCCGGCTTAGCCTTGTATGT	300	TGCCTTCCTTTAGCACAAATGTATCC	300	ACAGTGCACCAGGACCCATCACCA	50	0.93
Cat	TaqMan GEx (AB)						1.11
Cdkn2a	GAACGTCGCCCAGACCGAC	300	GCATCGCTAGAAGTGAAGCTAAGAAG	300	CCTGGAACTTCGCGGCCAATCCCAAGAG	50	1.00
c-Maf	GTGAGCAACGGCTTCCGAGA	300	GAGTGTCTTCTCTGTTCAGCTTCG	300	TCGAGCAGCGACAACCCCTTCCTCTCCC	50	0.96
Cxcl12	TAAACCAGTCAGCCTGAGCTA	300	TCAATGCACACTTGTCTGTTGTT	300	CGCCAGAGCCAACGTCAAGCATCTGA	50	0.99
Dnmt3a	GTCATGTGGTTCGGAGATGGC	50	CTGGAGGACTTCGTAGATGGCT	300	TGTGTGGAGAAGCTCATGCCGCTGAG	50	0.94
Dnmt3b	GTGAGACCAAGGACACCAGGAC	300	GTACTCCTGCACATGGTGGC	300	CGAACCCGACATAGCAATGGGACCTCCAG	50	1.05
Gcg	AGCACGCCCTTCAAGACA	900	CATCTCATCAGGGTCCTCATGC	900	ACCCCAGATCATTCCCAGCTTCCCAG	50	1.00
Igfbp4	GAAATCGAAGCCATCCAGGAAAG	300	GATCTTCATCTTGCTCCGATCTCT	300	CCCTGCAGTGCCCATGATCACAGGT	50	0.91
Ins1	TGGAACAACTGGAGCTGGGAG	300	GTAGAGGGAGCAAATGCTGGT	300	ACGCTTCTGCCGGGCCACCTCCAA	50	1.06
Itih5	GACATCCTACGTCTGGTCTCTG	50	ATGGTGATGGTGCGGAAGTAG	300	ACTCTGGTGTGACTGTGAATGGCGAGC	50	0.99
Ldha	CTGGGAGAACATGGCGACTC	300	GGTTAAGAGACTTCAGGGAGACG	300	TGCCTGTGTGGAGTGGTGTGAATGTTGC	50	1.06
Lmo4	CAAGTGCTCCTGCTGCC	300	GAGATGATACACGTTGCCTTGG	300	AGCGGTGCTTGCAGTGCCTGTGG	50	0.99
MafA	CTGGTATCCATGTCCGTGC	300	CGGTTCTTGAGCGTGCG	300	TCAGCAAGGAGGAGGTCATCCGAC	50	1.03
Oat	ATGCCATTGTCATCAGAGAAACC	50	CAGAAGCCCGTTATCTCGAAG	300	AGACTGTGATGCTTGGAAGGTGTGCCTG	50	1.00
Pdgfra	AACCTCAGCGTTGTGGC	50	ATCACCAACAGCACCAACAC	300	CTCCCACTCTGCGATCTGAACTCACAG	50	1.09
Slc16a1	GCTTGGTGACCATTGTGGAATG	50	CCCAGTACGTGTATTTGTAGTCTCC	900	CCCTGTCCTCCTAGGGCCACCACT	50	1.01
Slc2a2	ATCCCTTGGTTCATGGTTGCTG	900	TCCGCAATGTACTGGAAGCAG	900	TCCTACGGCTCTGGCACTGGCTG	50	0.97
Slc30a8	CTTCATCTTTATGGTGGCAGAG	300	CAAATGTCAGCCGCTTGGAAG	900	TTGCTGGGAGTCTGGCTATCCTCAC	50	1.03
Smad3	TTCCCTGCTGGCATTGAGC	300	GGTTCATCTGGTGGTCACTGG	300	CACCTCCTGGCTACCTGAGTGAAGATGG	50	0.90
Tet1	CAGACCGAAGATGTACCCTCAAC	300	GAAATACATGCTCCACGAACAGC	300	CGTACCTGCACCTGTCAAGGCATCGAC	50	1.02
Top2a	TaqMan GEx (AB)						1.17
Zfp36l1	ACACACCAGATCCTAGTCCTTGC	300	TGGGAGTGCTGTAGTTGAGCATC	300	ACCACCACCCTCGTGTCCGCC	50	0.98
Zyx	GCTGATGCAGGACATGGAACA	300	CAGGTGATGTGGAACAGTTGTC	300	AGGCAGAGCGTGGCAGTGAATGAGTCC	50	1.02
