# Supplementary material for: How stable is repression of disallowed genes in pancreatic islets in response to metabolic stress?
Source: PLoS One. 2017 Aug 9;12(8):e0181651. doi: 10.1371/journal.pone.0181651 (PMC5549890; doi:10.1371/journal.pone.0181651)
Supplement: S2 Table — (PDF) [file pone.0181651.s002.pdf]

| Gene | Forward primer | Conc. (nM) | Reverse primer | Conc. (nM) | Probe (6-FAM)–(TAMRA) | Conc. (nM) | Amplicon site |
|---|---|---|---|---|---|---|---|
| ***Actb*** | CTTCTTTGCAGCTCCTTCGTTG | 300 | CCCTGCAGTGAGGTACTAGC | 900 | CCACACCCGCCACCAGGTAAGCAG | 50 | +29/+134 |
| ***c-Maf*** | GTGTGCACGTTCGAGCTTTC | 300 | CAGATGGGCTGCAGGAGA | 900 | CCGCTGGCCACCCAGCACAG | 50 | -133/-7 |
| ***Cxcl12*** | ATCAAAGGTCTCAGCACCCA | 300 | CCCTTCCAGTTCCCTACCTAC | 300 | CCTTCTTGGCGCCCTCAGTGTCCG | 50 | -163/-84 |
| ***Igfbp4*** | AAGCAACATCCCCTTCGTTTC | 300 | ACAACATCTGAAAGTCCTTTGCC | 300 | AGGCCACCGCTTCCAAACTGCGT | 50 | -312/-181 |
| ***Itih5*** | GGACAGAGAGGCGTGGC | 300 | CTTTATGCCCTTCAGGCCATC | 300 | TAGTGGTAGTGCGGCGACAGGC | 50 | -89/-12 |
| ***Oat*** | GGAAGGCAGCAGAACTTGGC | 50 | AGTTCATTGGTTAAGGCGGAGG | 300 | CGAGAGTGAGCCTCTTGTGATTGGTGTG | 50 | -116/-13 |
| ***Pdgfra*** | AGCTGGAAGGAAAGGGACTTG | 900 | CCACAAGATCGCCACCAGATA | 50 | AGGCGACACGGATCTACCCGACGAT | 50 | -207/-125 |
| ***Slc16a1*** | TTTCTCCTCCAGAGCCTGA | 900 | ACATCCTTATCAGCGCTCTGA | 900 | GGACGCCATCGTGGGCCTCA | 50 | -324/-233 |
