# Supplementary figures and images for: How stable is repression of disallowed genes in pancreatic islets in response to metabolic stress?
Source: PLoS One. 2017 Aug 9;12(8):e0181651. doi: 10.1371/journal.pone.0181651 (PMC5549890; doi:10.1371/journal.pone.0181651)

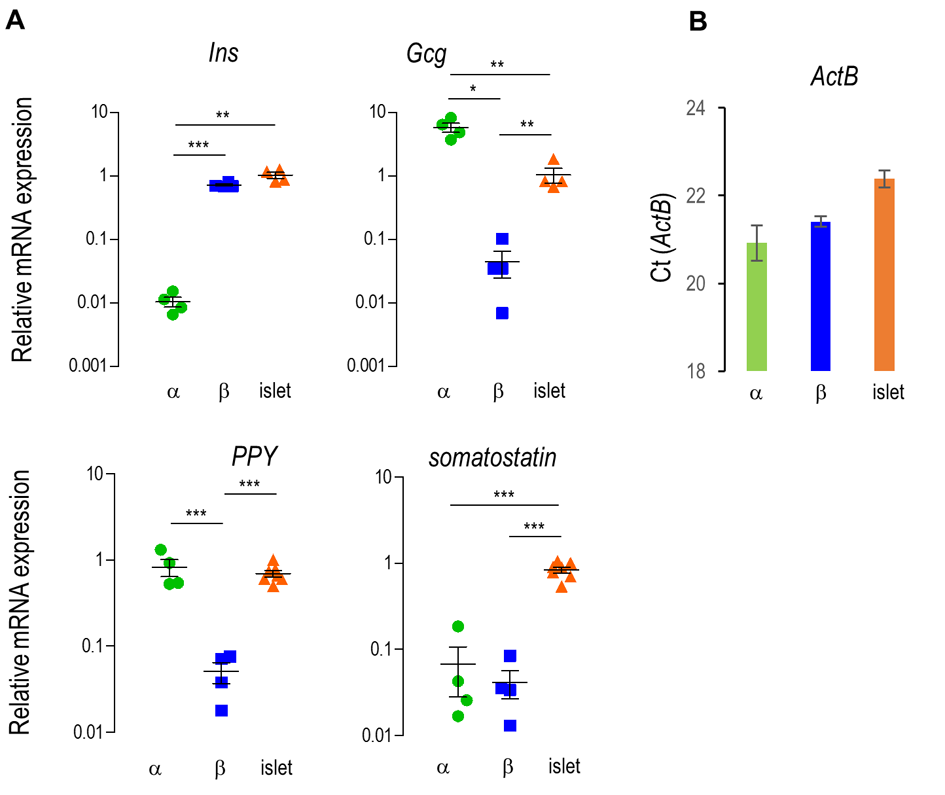

Supplement: S1 Fig — mRNA expression of insulin, glucagon, pancreatic polypeptide (PPY) and somatostatin in alpha-, beta- cells, compared with whole islets, measured via quantitative RT-PCR. The contamination of beta cells in the alpha cell preparations and vice versa was in the order of magnitude of 1 percent as estimated from the measurement of pancreatic islet hormones. Contamination with delta-cells (somatostatin) is in the same range. PPY expression in the beta-cell preparation is also in the order of magnitude of 1 percent. In contrast, the expression of PPY in the alpha-cells is as high as in islets, a phenomenon that has been noticed before (Gilon P., unpublished micro array data; [54,55]) and most probably is not a result of contamination with PP-cells. (B) Ct values of beta actin, used for normalization of the QPCR data. (TIF) [file pone.0181651.s003.tif]

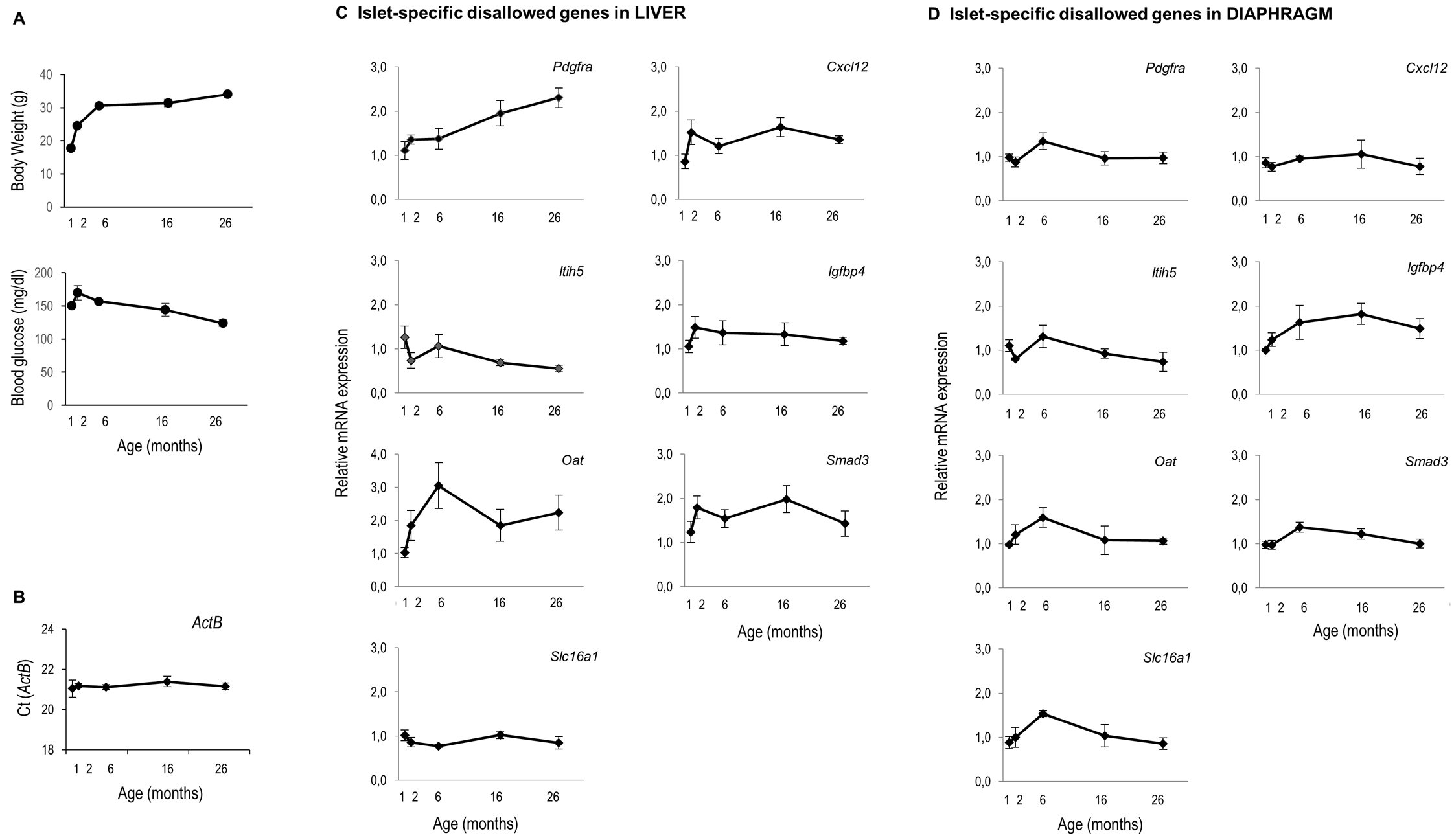

Supplement: S2 Fig — (A) Body weight and blood glucose levels of the used mice at different ages; (B) Ct values of beta actin, used for normalization of the quantitative RT-PCR data; (C, D) mRNA expression levels of the 14 islet specifically repressed genes in liver and diaphragm of mice at 5 different ages (1–2–6–16–26 months), measured via quantitative RT-PCR. Data are normalized for beta actin and expression at 1 month is set as 1. Data represent mean±SEM, N = 4. (TIF) [file pone.0181651.s004.tif]

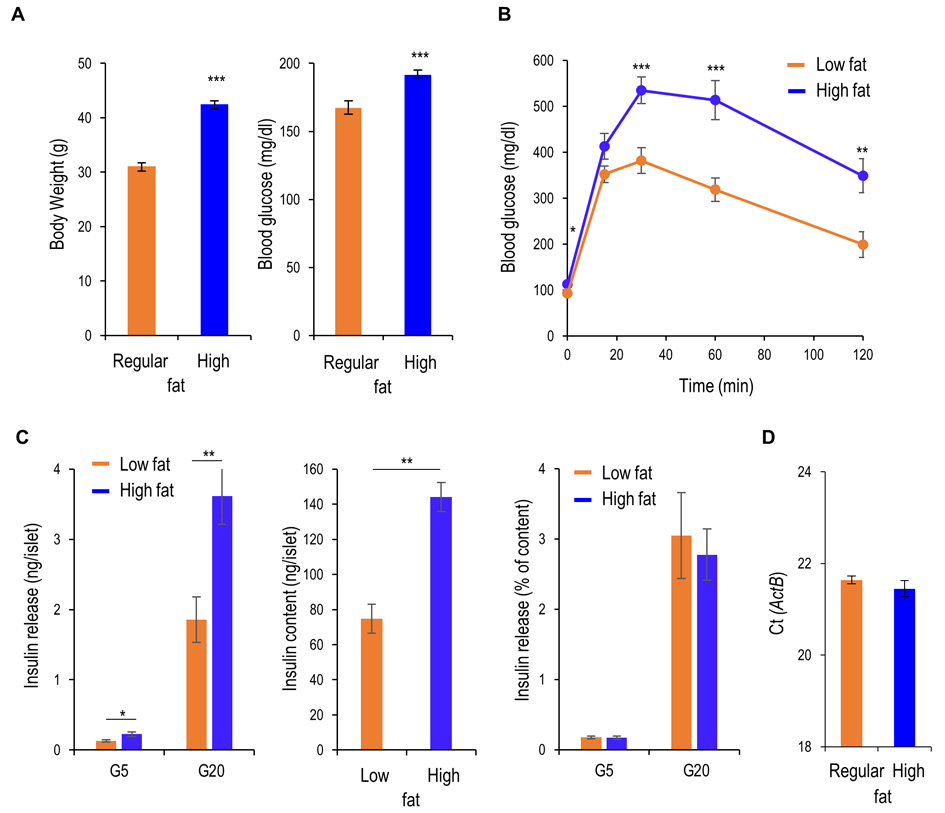

Supplement: S3 Fig — (A) Body weight and blood glucose levels of the analyzed mice after 16 weeks of a high fat diet; (B) intraperitoneal glucose tolerance test after 15 weeks on the specific diet, N = 8, 18 weeks old mice); (C) Glucose stimulated insulin release after 20 weeks on the specific diet (23 weeks old mice): release per islet (left panel), insulin content (middle panel) and release per content (right panel), G5 = 5 mM, G20 = 20 mM, N = 7; (D) Ct values of ActB, used as reference gene. N = 4. Data represent mean±SEM. Statistical analysis: student t-test with multiple comparison correction (Bonferroni). (TIF) [file pone.0181651.s005.tif]

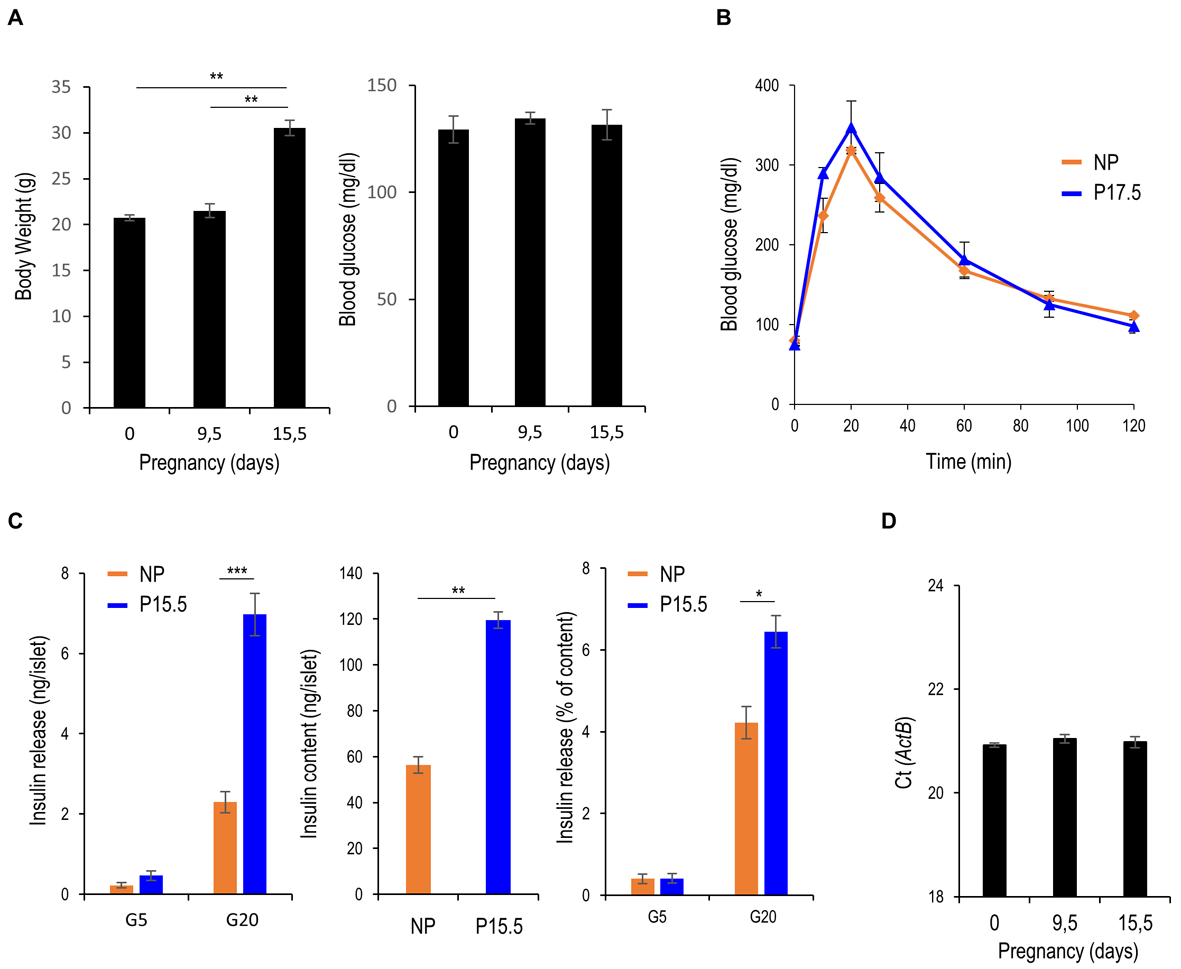

Supplement: S4 Fig — (A) Body weight and blood glucose levels during pregnancy (pregnancy day 0–9.5–15.5), (B) oral glucose tolerance test, N = 4, (C) Glucose stimulated insulin release: release per islet (left panel), insulin content (middle panel) and release per content (right panel), G5 = 5 mM glucose, G20 = 20 mM glucose, N≥5, (D) Ct values of ActB, used as reference gene. N = 4. Mice 12 weeks of age. Data represent mean±SEM. Statistical analysis: student t-test with multiple comparison correction (Bonferroni). (TIF) [file pone.0181651.s006.tif]

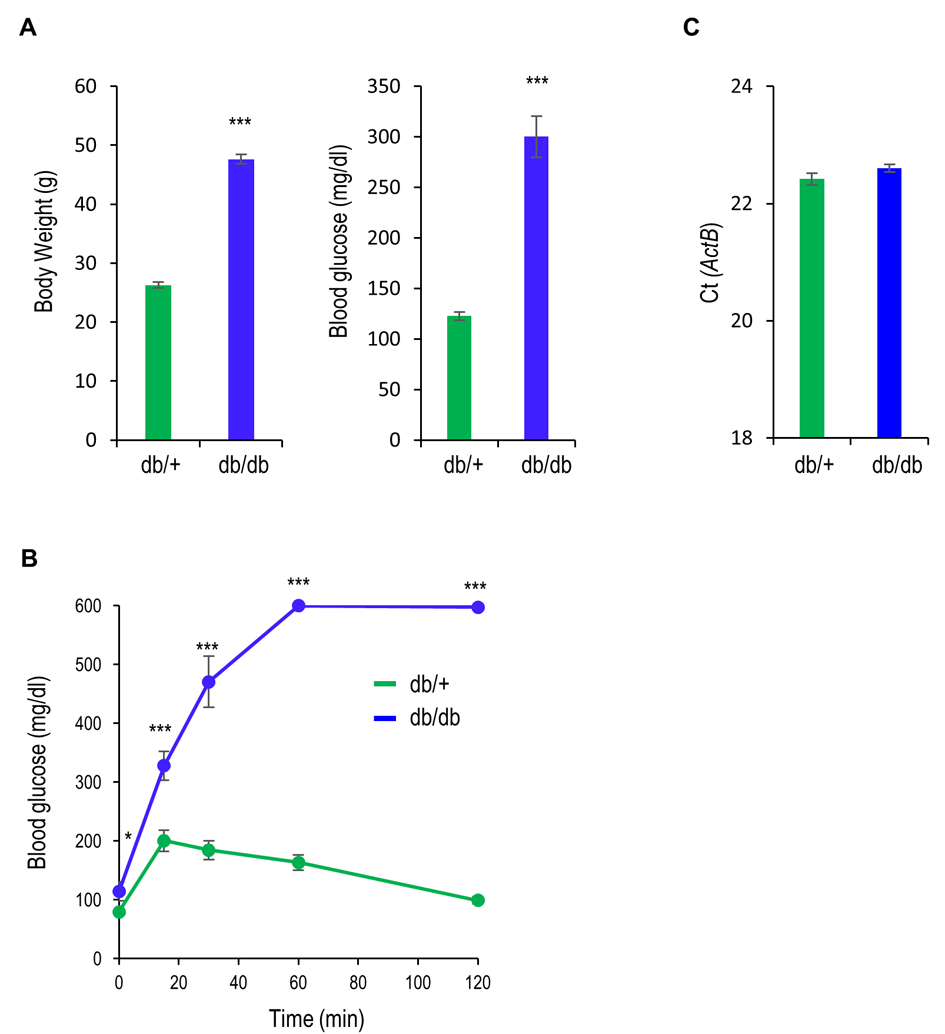

Supplement: S5 Fig — (A) Body weight and blood glucose levels of 16 weeks old db/db and control db/+ mice, N = 8. (B) Intraperitoneal glucose tolerance test at the age of 15 weeks, N = 8. (C) Ct values of ActB, used as reference gene. N = 7–8. Data represent mean±SEM. Statistical analysis: student t-test. (TIF) [file pone.0181651.s007.tif]
